# A robust audio steganography technique based on image encryption using different chaotic maps

**DOI:** 10.1038/s41598-024-70940-3

**Published:** 2024-09-27

**Authors:** Marwa A. Nasr, Walid El-Shafai, El-Sayed M. El-Rabaie, Adel S. El-Fishawy, Heba M. El-Hoseny, Fathi E. Abd El-Samie, Nariman Abdel-Salam

**Affiliations:** 1 Department of Electronics and Electrical Communication Engineering, Faculty of Electronic Engineering, Menoufia University, Menouf, 32952 Egypt; 2 Security Engineering Lab, Computer Science Department, Prince Sultan University, 11586 Riyadh, Saudi Arabia; 3 Computer Science Department, Higher Future Institute for Specialized Technological Studies, Cairo, 3044 Egypt; 4 Department of Information Technology, College of Computer and Information Sciences, Princess Nourah Bint Abdulrahman University, 11671 Riyadh, Saudi Arabia; 5 Electronics and Communications Engineering Department, Faculty of Engineering, Canadian International College (CIC), Giza, Egypt

**Keywords:** Chaos-based cryptography, Image encryption, Information security, Audio steganography, Engineering, Mathematics and computing

## Abstract

The development of innovative methods for concealing critical data in multimedia files has exploded in information security in recent years. Cryptography and steganography cannot be used alone to protect data; rather, they can be combined and used in a single system. Audio steganography is among the most important information security techniques. It involves the concealment of information within audio signals to achieve covert communication. This paper introduces a comprehensive technique that integrates chaos Henon, Baker, and Arnold maps for image encryption with audio steganography to create a robust and secure audio steganography technique. First, the target image is encrypted using chaotic maps. Then, it is embeded within the high frequencies of the cover audio signal based on the Inverse Short Time Fourier Transform (ISTFT) to be transmitted to the destination through the channel. By integrating both encryption and concealment techniques, the cover audio signal quality can be preserved. Moreover, the hidden image security and robustness are improved, making the technique resistant to many types of attacks. The simulation results confirm that the suggested technique is robust in the presence of attacks. It achieves a distinct perceptual quality with an appreciated peak signal-to-noise ratio (PSNR) of 91.2 dB and a Mean Square Error (MSE) of 7.5 × 10^–10^. The randomness of the resulting encrypted image has successfully passed the National Institute of Standards and Technology (NIST) statistical test suite.

## Introduction

Cryptography and steganography stand out as crucial techniques in ensuring data security. Cryptography plays a vital role in transforming data into encrypted forms, rendering it unintelligible to unauthorized individuals. Both steganography and cryptography serve the purpose of safeguarding confidential data. However, their approaches diverge. Steganography involves concealing data in such a way that its presence remains undetectable, while cryptography focuses on encoding data to prevent comprehension by unauthorized parties. The act of integrating data into another digital entity covertly, without revealing its existence, characterizes steganography.

Steganography is a type of secret communication for data concealment in which a secret message such as a text message, an image, or a video can be concealed within the cover data. It is more different than cryptography, which is defined as covered writing, and less common than it^1,2^. Audio steganography is a type of data concealment in which secret information is hidden within a cover audio signal such as MP3, WAV, and AU files, without causing noticeable alterations to audio signals. The secret message can take any format, including text, image, audio, and video^3,4^. The most common hiding techniques for audio steganography are insertion-based, substitution-based, and generation-based techniques. Spread spectrum, echo concealment, Least Significant Bit (LSB), and phase coding are the most utilized methods for audio steganography^1^. The ability of audio steganography to covertly hide the secret information in the cover audio has made it more popular in the multimedia context^5^.

Image encryption is a crucial aspect of securing digital content, ensuring the confidentiality and integrity of visual data^6^. This process involves applying mathematical algorithms to transform the pixel values of an image into a scrambled or encrypted form, rendering it unreadable without the appropriate decryption key. The main goal is to protect sensitive visual information from unauthorized access or tampering during transmission or storage^7^. By implementing robust image encryption methods, organizations and individuals can safeguard their visual data against potential threats and maintain their privacy^8^.

Most traditional encryption techniques, like the Advanced Encryption Standard (AES), the International Data Encryption Algorithm (IDEA), and the Data Encryption Standard (DES)^9^, have low speeds and are therefore not appropriate for real-time usage because of high data volumes, strong adjacent pixel correlations, and other intrinsic characteristics of the image such as higher redundancy. The chaos theory is continually applied in modern cryptography.

Chaos can be utilized for building cryptosystems that meet the usual Shannon requirements of confusion and diffusion, since it operates randomly. Moreover, it is vulnerable to initial conditions and parameter changes. It has been demonstrated that the characteristics of chaos-based techniques are exceptionally satisfied in numerous areas of interest, such as processing power, complexity, speed, security, and computational complexity. So, the systems for encrypting images using chaos theory are considered superior and more resilient to attacks^10^. Chaotic map states generated by iterative processing are used to achieve characteristics such as high unpredictability, balancedness, confusion, and diffusion required in classical cryptography techniques. Because of its complicated dynamics and ergodicity, chaos seems to be an excellent choice for cryptography^11,12^.

**Fig. 1:**
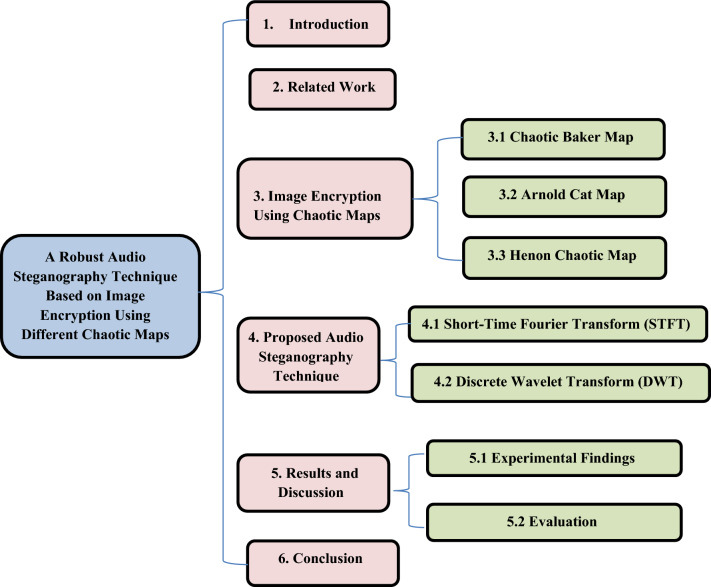
A flowchart for the presented paper.

While extensive investigations have been conducted on audio security and data transmission, the potential for intruders to uncover novel methods for accessing sensitive information persists. This presents a distinct risk of data compromise through specific avenues. The current measures for safeguarding the security of Internet-based data exchange and privacy of users are inadequate, thereby allowing unauthorized interception. Consequently, a pressing need exists to enhance the security and confidentiality of data transfer. To address this imperative, a range of techniques, such as steganography and cryptography, can be employed. The flowchart for the presented paper is shown in Fig. 1.

**Fig. 2:**
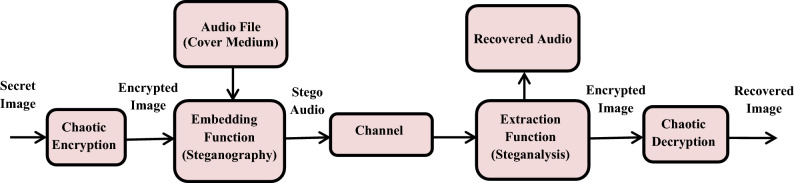
Flowchart for embedding the encrypted image within the cover audio signal.

Although encryption and steganography provide some protection for data, this research presents a combination of two techniques into a unified system to protect confidential information. There are two levels of data protection in this system for both image and audio hiding. The images are encrypted using a chaotic map method, and then hidden inside the high frequencies of the audio file based on the Short-time Fourier transform (STFT) technique, since the human auditory system is not very sensitive to audio at high frequencies. Preserving the message validity, integrity, and security are the primary goals of this strategy. The flowchart for embedding the encrypted image into the audio signal is shown in Fig. 2. We use audio signals at a suitable size at a 44.1 kHz sampling frequency and a set of images. To assess the technique effectiveness, a comparison study is included.

### Contributions and motivation

While extensive investigations have been conducted on audio security and data transmission, the potential for intruders to uncover novel methods for accessing sensitive information persists. This presents a distinct risk of data compromise through specific tools. The current measures for safeguarding the security of Internet-based data exchange and security of users are inadequate, thereby allowing unauthorized interception. Consequently, a pressing need exists to enhance the security and confidentiality of data transfers. To address this imperative, a range of techniques, such as steganography and cryptography, can be employed.

This paper introduces a comprehensive technique that integrates chaos Henon, Baker, and Arnold maps for image encryption with audio steganography to allow robust and secure audio steganography. The suggested technique depends first on encrypting the target image using a chaotic map. Then, it is embeded within the high frequencies of the cover audio signal based on the ISTFT to be transmitted to the destination through the channel. By integrating both encryption and concealment techniques, the cover audio signal quality can be preserved, and the hidden image security and robustness are improved, making the technique resistant to many types of attacks.

The primary contributions of this work are:

- Performing secret image encryption with chaotic maps.
- Generating spectrograms from the encrypted images.
- Using the ISTFT to convert image spectrograms into audio-like signals.
- Embedding encrypted images within the cover audio files.
- Evaluating the performance of the suggested technique using different performance evaluation metrics.
- Conducting comprehensive comparisons of the various stages of the suggested technique with existing works.

This research is motivated by the need to enhance the security of data transmission in multimedia contexts. Traditional methods often fall short in terms of speed and resilience to attacks, necessitating the development of more robust solutions. The proposed technique leverages the strengths of chaotic maps for encryption and advanced steganography methods to achieve superior security and imperceptibility.

The rest of this paper is organized as follows. Section "Related work" provides an overview of the related work. In Sect. "Image cryptography using chaotic maps", the image encryption techniques are discussed. In Sect. "Proposed audio steganography technique", the proposed audio steganography technique is explained. Section "Results and discussion" presents the results and discussions. Section "Conclusion and future work" provides the conclusion.

## Related work

Research on audio steganography techniques has been conducted in a wide range of papers. Most of the previous research has focused on various methodologies, including frequency-domain techniques and spatial-domain techniques. The use of image encryption algorithms combined with steganography has been explored to enhance system security. However, the combination of different chaos map image encryption techniques with STFT steganography in audio signals has not been extensively studied.

Nouf et al.^13^ combined audio-based steganography with the Rivest–Shamir–Adleman (RSA) cryptography in two successive layers to create an enhanced method for protecting critical texts on personal computer systems. Capacity versus security trade-off is demonstrated in the study, giving customers a choice between different LSB approaches.

The increasing need for secure information transmission due to rising cases of data theft has been discussed by Shambhu et al.^14^. They introduced an audio steganography approach that depends on embedding a secret message within digital audio. It enhanced the security against various attacks, while maintaining imperceptibility, with evaluation scores close to the highest values in the absence of attacks.

Sazeen et al.^15^ presented a novel steganography technique that securely hides compressed images within audio files using the LSB algorithm for WAV format and preserved the audio quality. A contourlet transform and duffing oscillation have been employed in the concealment algorithm described by Abbas^16^ to improve security, concealment ability, and transparency when delivering sensitive spoken messages.

Pranati et al.^17^ discussed the growing importance of securing sensitive data transmitted over public networks through cryptography and steganography. They presented a system that enhances security by combining audio steganography with visual cryptography by dividing the secret image into subparts and hiding it within audio files, making interception difficult, and ensuring a two-step security mechanism.

Enas et al.^18^ described the development of an efficient system for secure communication of audio signals by transforming them into a format similar to RGB images and concealing them in cover audio files using LSB and Discrete Wavelet Transform (DWT) techniques.

Pooja^19^ presented a method that combines AES encryption and LSB data hiding. The integrated approach enhances data security by encrypting information before concealing it within images or audio files, resulting in high-quality stego-images and audio with improved security metrics. Anthony et al. ^20^ presented an audio steganography algorithm that depends on embedding an authentication key into sound, aiming to enhance both usability and security in audio-based systems.

Salamudeen et al.^21^ discussed the effectiveness of secure communication through covert methods, specifically focusing on steganography to conceal secret messages within audio samples. This method involves the twin K-Shuffling and embedding approach, providing 3-layer protection for private data, and satisfying the encryption and insertion demands of the concealment systems.

Piotr et al.^22^ investigated the suitability of Commercial Off-The-Shelf (COTS) software for audio steganography, which is a technique used to conceal and transmit sensitive information. The analysis revealed that it is possible to detect hidden messages in audio files. It provided valuable insights for hacking attack detection and prevention.

## Image cryptography using chaotic maps

Chaos-based algorithms are an excellent alternative for both secure communication and cryptography because they provide a strong combination of high processing capacity, acceptable computational overheads, low complexity, excellent security, and high speed. Chaos-based encryption algorithms are thought to be helpful in real-world applications, among other advantages. One of the most important uses of chaos theory is in the field of chaotic communications, which aims to secure information transmitted through a communication medium. To allow secure communications, one must be aware that the message content is not available to potential listeners^23^.

Chaotic systems offer diverse and dynamic characteristics that underpin security, or privacy, in chaotic communications. Data is encoded using several characteristics of chaos dynamics, including spread spectrum, noise-like dynamics (pseudorandom noise), and complicated behavior^24^. Different kinds of chaotic maps exist. In this research, we study the effect of using several two-dimensional chaotic maps, including the Baker, Arnold, and Henon maps, and a combination of them together in encrypting images, as a preliminary stage to include them within an audio envelope signal to strengthen the protection of the suggested audio steganography technique and make it challenging for attackers to discover the messages. We will review the encryption methods used below.

### Chaotic Baker map

The image processing community is familiar with the chaotic Baker map as an encryption technique. It depends on a secret key to randomly arrange pixels in a square matrix with a size of *N* × *N* based on permutation^25^. An effective method for randomly assigning values to entries in the square matrix is the heart of discretized Baker map. Let *B*(*n*_*1*_,…, *n*_*k*_) be the discretized map, and let [*n*_*1*_….., *n*_*k*_] be the vector corresponding to the secret key, *S*_*key*_. The total number of data elements in a row is denoted by *N.* The *S*_*key*_ is selected, so that *n*_1_ + ….. + *n*_*k*_ = *N* and each integer *n*_*i*_ divides *N*. Assume *N*_*i*_ = *n*_1_ + ….. + *n*_*i*-1_. The data item is relocated to the indices (*r*, *s*):

1 $$B_{{(n_{1} ,...,n_{k} )}} (r,s) = \left[ {\frac{N}{{n_{i} }}\left( {r - N_{i} } \right) + s\,\bmod \left( {\frac{N}{{n_{i} }}} \right),\frac{{n_{i} }}{N}\left( {s - s\,\bmod \left( {\frac{N}{{n_{i} }}} \right)} \right) + N_{i} } \right]$$

where *N*_*i*_ ≤ *r* < *N*_*i*_ + *n*_*i*_, 0 ≤ *s* < *N,* and *N*_1_ = 0.

**Fig. 3:**
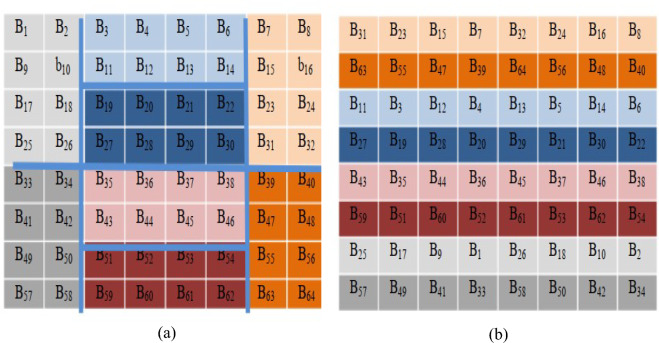
An 8 × 8 matrix with chaotic Baker interleaving. (**a**) The 8×8 matrix. (**b**) The matrix chaotic interleaving result.

A square matrix of dimensions *N* × *N* is used to create* N* rectangles with width *n*_*i*_ and number of components *N.* The elements of each rectangle are rearranged to form rows within the permuted rectangle. Rectangles are arranged in descending order, beginning with the larger values. The scan inside each rectangle starts at the lower left corner and moves up towards the upper parts. An illustration of the chaotic interleaver of an 8 × 8 matrix (i.e., *N* = 8, *S*_*key*_ = [*n*_1_, *n*_2_, *n*_3_] = [2, 2, 4]) is presented in Fig. 3.

### Arnold cat map

A confusion map called Arnold's Cat Map (ACM) holds Vladimir Arnold's name. It improves the image security. The original pixel layout of the image, which is not always a cat, is altered randomly. The map period is consistent. Unfortunately, there are two major drawbacks of ACM. Sometimes the original image can be restored. Furthermore, as the pixel values remain unchanged, the encoded image histogram is identical to the original image histogram^26^. The ACM is used to change the pixel positions of the base image. Suppose that the original image dimensions are *N* $\times$ *N*, the 2-D ACM is defined as follows:

2 $$\left[ \begin{gathered} x_{m + 1} \hfill \\ y_{m + 1} \hfill \\ \end{gathered} \right] \, = {\text{ A }}\left[ \begin{gathered} x_{m} \hfill \\ y_{m} \hfill \\ \end{gathered} \right]\left( {\text{mod}}\,N \right) = \, \left[ \begin{gathered} {\text{1 }}\,\,\,\,\,\,\,\,\,\,\,\,p \hfill \\ q \,\,\,\,\,\,\,\,\,\,\,\,\,pq + 1 \hfill \\ \end{gathered} \right]\left[ \begin{gathered} x_{m} \hfill \\ y_{m} \hfill \\ \end{gathered} \right]\left( {\text{mod}}\,N \right)$$

where det (*A*) = 1, *p* and *q* are positive integers. After running the ACM once, the initial pixel position (*x*_*m*_, *y*_*m*_) becomes (*x*_*m*+1_, *y*_*m*+1_), where *m* = 0,1, 2, 3, …. There are positive integers *R*, such that (*x*_*m*+1_, *y*_*m*+1_) = (*x*_*m*_, *y*_*m*_) after iterating *R* times. The original image size *N*, along with the parameters *p* and *q*, determine the time *R*. Consequently, *p*, *q*, and *R* hold the secret keys. Using the ACM, it is incredibly efficient to change the pixel positions, because there is simply a linear transformation and mod function. After many rounds, it is possible to disrupt the association between neighboring pixels.

### Henon chaotic map

Henon chaotic map is described as follows^27^:

3 $$\begin{array}{*{20}l} {x_{i + 1} = 1 - ax_{i}^{2} + y_{i} ,} \\ {y_{i + 1} = bx_{i} } \\ \end{array} {,}\quad \quad i = 0,1,2,.......$$

A basic two-dimensional map with quadratic nonlinearity is presented by the extensively researched Henon map. This map provided the first illustration of the odd attractor with a fractal structure. It lends itself readily to numerical research because of its simplicity. There were a lot of computer studies that came after. However, the whole image of all conceivable bifurcations resulting from altering the parameters *a,* and *b* is still far from being completed. The system is chaotic if one chooses *a* = 0.3, and *b* = 1.4. There are three steps in the encryption process, which are shown as follows:

**Step 1:** The 2-D chaotic map is converted into a 1-D chaotic map^28^:

4 $$x_{i + 2} = 1 - ax_{i + 1}^{2} + bx_{i}$$

in which *a* = 0.3, and *b *$\in$[1.07,1.4]. The initial values *x*_0_, *x*_1_, *a*, and *b* might all be used to represent the key.

**Step 2:** The image pixel values are altered using the Henon map. First, formula (3) yields the Henon chaotic map. A matrix is then created using the pixel data.

**Step 3*:*** The exclusive OR operation between the image pixel values and the transform matrix of those values is carried out bit by bit. The outcome is the encrypted image^29,30^. The chosen parameters are *x*_0_ = 0.01, *x*_1_ = 0.02, *a* = 0.3, and *b* = 1.4.

The chaotic maps have several advantages, when it comes to security applications:

- **Sensitivity to initial conditions:** Chaotic maps are highly sensitive to initial conditions, meaning a small change in the input can lead to drastically different outputs. This property makes them useful in encryption schemes, where even a minor change in the key or plaintext can result in a completely-different ciphertext.
- **Pseudo-randomness:** Chaotic maps exhibit a behavior that appears random, making them suitable for generating pseudorandom sequences. These sequences can be used for tasks, such as generating cryptographic keys or initialization vectors in secure communication protocols.
- **Nonlinearity:** Chaotic maps are inherently nonlinear, meaning that their behavior cannot be easily predicted or modeled using linear equations. This nonlinearity adds an additional layer of complexity to cryptographic algorithms, making them more resistant to attacks.
- **Complex dynamics:** Chaotic maps often exhibit a complex dynamic behavior, represented in bifurcations, period-doubling cascades, and strange attractors. Exploiting these complex dynamics can enhance the security of cryptographic systems by creating intricate patterns that are difficult to decipher without knowledge of the system parameters.
- **Low complexity:** Some chaotic maps have relatively-simple mathematical descriptions and low computational complexity, making them suitable for implementation in resource-constrained environments, such as embedded systems or IoT devices.
- **Key-space expansion:** Chaotic maps can be used to expand the key space of cryptographic algorithms by introducing additional randomness into the encryption process. This makes brute-force attacks more difficult, as the search space becomes larger due to the chaotic nature of the system.
- **Resistance to cryptanalysis:** Chaotic maps can be resistant to various cryptanalytic attacks, including differential and linear cryptanalysis, due to their complex and unpredictable nature. This makes them attractive candidates for building secure encryption and authentication schemes.

## Proposed audio steganography technique

An effective data security technique is presented, relying on integrating two different security techniques, to build a more secure and resistant system to various attacks and unauthorized operations. It depends on encrypting the secret images, converting them into audio-like signals, and then hiding them in the high frequencies of the audio signals to increase the strength of the technology secrecy.

At the transmission end, firstly, the secret image is encrypted using different chaotic maps such as Henon, Arnold, and Baker chaotic maps. The encrypted secret image is then flipped twice to create two identical images, and we use ISTFT to convert them into an audio-like signal, to perform random permutation. Secondly, DWT is applied to the audio signal as a means of continuous cutting, where the high frequencies of the carrier are identified, and the part closest to the secret message is found. The converted image is then embedded within high carrier frequencies and sent through the channel. After that, the Inverse Discrete Wavelet Transform (IDWT) is used to get the stego-audio signal.

**Fig. 4:**
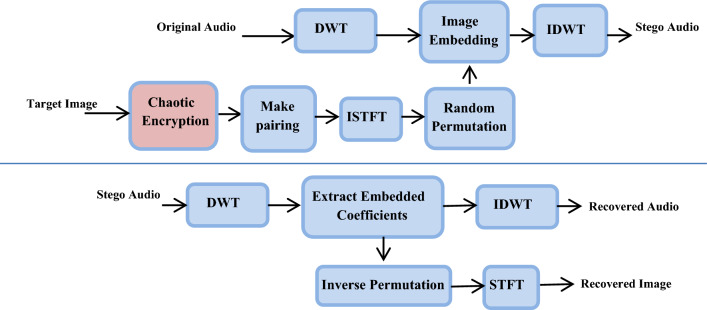
Proposed technique for audio steganography based on image encryption using chaotic maps.

The image pairing process is done for vertical symmetry, as the image would be complex if converted directly to an audio-like stream via ISTFT. In this scenario, recovering the image from the audio signal will be a major challenge. Naturally, the digital signal frequency-domain version is uniformly symmetric. As a result, symmetry must be considered in the frequency spectrum. Figure 4 shows the block diagram for the proposed technique.

At the receiving end, the embedded image can be extracted by applying DWT to analyze the audio signal and image, and extract the hidden converted image again. We can also reverse the corresponding permutation used at the transmission end using the same key. Then, we apply the STFT and chaotic decoding to extract the target image. We can also apply IDWT to extract the original audio signal of the envelope.

### Short-time Fourier transform (STFT)

**Fig. 5:**
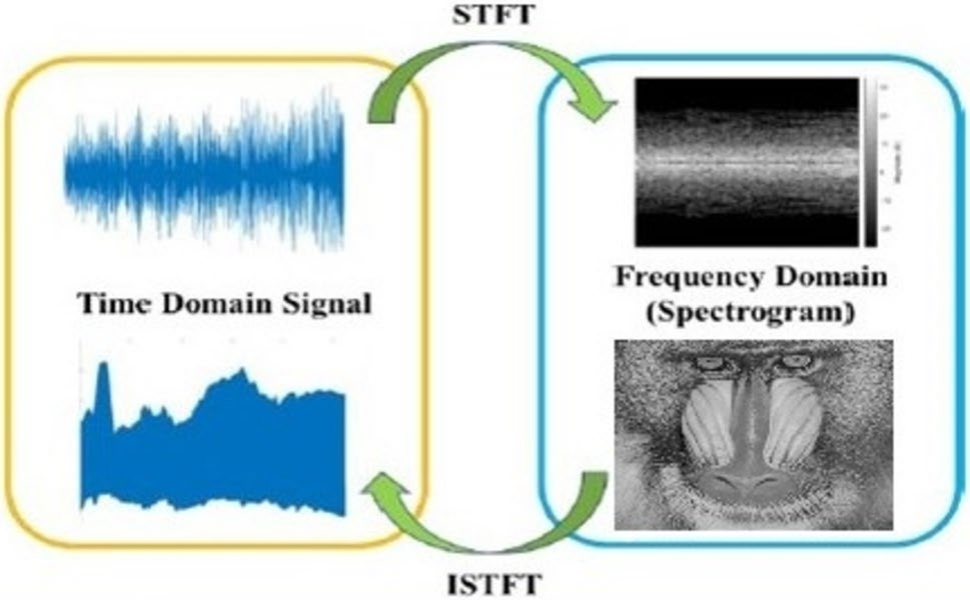
STFT of the speech signal and ISTFT of the image^31^.

The STFT serves to analyze the frequency and phase characteristics of signal components within localized time intervals^31^. It involves partitioning a continuous signal into shorter segments of uniform duration, and then computing the Fourier transform separately for each segment to reveal its frequency content. Each segment yields its own Fourier spectrum. Figure 5 illustrates a conceptual representation of the STFT applied to speech signals and the ISTFT applied to images.

The mathematical expression of the STFT is as follows:

5 $$X\left[ {k,n} \right] = \mathop \sum \limits_{m = 0}^{L - 1} w\left[ m \right]x\left[ {n + m} \right]e^{{ - j\left( {\frac{2\pi k}{N}} \right)m}}$$

In this context, we represent the input signal as *x*[*n*] and the window function as *w*[*m*]. Specifically, we employ a Kaiser window in this scenario, characterized by the following mathematical expression:

6 $$w\left[ n \right] = \left\{ {\begin{array}{*{20}l} {\frac{{I_{0} \left[ {\pi \alpha \sqrt {1 - \left( {\frac{2n}{N} - 1} \right)^{2} } } \right]}}{{I_{0} \left( {\pi \alpha } \right)}},} & {0 \le n \le N} \\ {0,} & {{\text{otherwise}}} \\ \end{array} } \right.$$

In the context of our study, we denote the zero^th^-order modified Bessel function as *I*_0_, where *N* + 1 represents the duration of the window, and α is a non-negative real parameter that governs the window shape.

### Discrete wavelet transform (DWT)

**Fig. 6:**
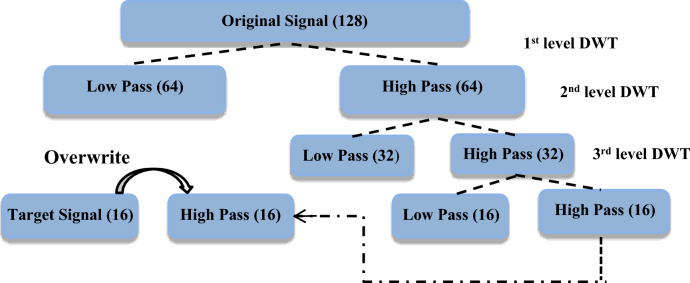
The DWT of an audio signal^31^.

The DWT denotes a wavelet transformation method employing discretely-sampled wavelets within the realms of numerical and functional analysis. Distinguished by its capacity for temporal resolution, DWT stands apart from Fourier transform and alternative wavelet transformations by adeptly capturing both frequency and positional data, particularly regarding temporal location. This concept is visually demonstrated in Fig. 6, showcasing the DWT applied to an audio signal^31^.

## Results and discussion

### Experimental findings

The proposed technique was tested using appropriately-sized recorded audio signals in the .mp3 and .wav formats, with sample rates of 44.1 kHz. With MATLAB, all coding, simulations, and analysis were carried out. Tables 1, 2, 3, 4 display the system performance evaluation results.

**Table 1:** Recovered images and evaluation metrics for the audio steganography technique using different chaotic maps for encrypting the embedded image.

Image encryption technique	Recovered image				Encrypted image			
	Recovered Image	PSNR (dB)	SSIM	Correlation	PSNR (dB)	SSIM	Correlation	NPCR
No Encryption		36.6777	0.9654	0.9965	–	–	–	–
Henon		11.5719	0.0493	0.38057	9.4115	0.0115	0.0061	0.9958
Arnold		18.7285	0.6930	0.7574	12.0231	0.0281	0.0015	0.9929
Baker		18.0895	0.2104	0.7114	12.0721	0.0276	0.0126	0.9892
Henon + Arnold		11.5565	0.04756	0.3787	9.4069	0.0100	0.0044	0.9961
Henon + Baker		11.5579	0.0475	0.37975	9.3790	0.0100	0.0008	0.9960
Arnold + Baker		18.0444	0.20658	0.70778	12.0310	0.0262	0.0032	0.9931

**Table 2:** Evaluation metrics of stego-audio signals.

Image encryption technique	PSNR (dB)	SNR	MSE	SNR_seg_	SD (dB)	LLR
No encryption	91.2183	72.2355	7.5538 × 10^–10^	71.3321	0.005	4.9873 × 10^–4^
Henon						
Arnold						
Baker						
Henon + Arnold						
Henon + Baker						
Arnold + Baker

**Table 3:** Comparison of the proposed technique with a related one.

Encryption technique	References	Correlation	Maximum deviation	Irregular deviation	Time (s)
Henon	^32^	0.0062	185,188	186,122	0.7482
	The proposed system	0.006174	185,188	186,122	1.0015
Arnold	^32^	0.0015	0	256,952	2.76
	The proposed system	0.00147	0	256,952	1.5
Baker	^32^	0.0710	1.252 × 10^5^	162,240	4.8859
	The proposed system	0.012679	0	520,192	1.37
Henon + Arnold	^32^	–	–	–	–
	The proposed system	0.0044	186,070	186,066	2.523
Henon + Baker	^32^	–	–	–	–
	The proposed system	8.835 × 10^–4^	185,819	185,208	2.27
Arnold + Baker	^32^	–	–	–	–
	The proposed system	0.00329	0	256,960	2.8396

**Table 4:** Comparison between the proposed audio steganography technique and some commonly-used techniques.

	LSB coding	Phase coding	Spread spectrum	Proposed technique
Image				
MSE	0	4.7876 × 10^–7^	1.6909 × 10^–4^	2.1489 × 10^–4^
PSNR (dB)	Inf	63.1988	37.7189	36.677
SSIM	1	0.9999	0.9739	0.9654
Audio				
MSE	8.3212 × 10^–9^	9.2808 × 10^–7^	1.25 × 10^–13^	7.5 × 10^–10^
SNR	61.2462	41.5323	110.39	72.2578

#### Experimental conditions

##### Hardware and software setup

- Hardware: The experiments were conducted on a PC with an Intel Core i7 processor, 16 GB RAM, and a 1 TB SSD.
- Software: MATLAB R2023a was used for all coding, simulations, and analyses. The Image Processing Toolbox and Signal Processing Toolbox were extensively utilized.

##### Data preparation

- Audio Signals: A dataset of 50 audio files in .mp3 and .wav formats, each with a duration of 30 s, was used. The audio files were sampled at 44.1 kHz.
- Images: A set of 100 grayscale images with dimensions of 256 × 256 pixels was used for the secret images to be embedded within the audio signals.

#### Image encryption procedures:

- Henon Chaotic Map:
  - Initialization: The Henon map depends on two parameters, *a* and *b*, with typical values of *a* = 1.4 and *b* = 0.3. Initial conditions for *x* and *y* are set.
  - Iteration: For each pixel value in the image, the Henon map equations are iterated as in Eq. (3).
  - Permutation: The pixel values are rearranged according to the generated chaotic sequence.
- Arnold Cat Map (ACM):
  - Initialization: The ACM is defined for a square image of size *N* × *N*.
  - Transformation: Each pixel position is transformed using the ACM of Eq. (2).
  - Iterations: The process is repeated for several iterations to achieve the desired level of scrambling.
- Baker Chaotic Map:
  - Initialization: The Baker map is applied to a square image matrix of size *N* × *N*.
  - Transformation: The image matrix is divided into vertical strips, which are then rearranged:
    7 $$B\left(x,y\right)=\left\{\begin{array}{ll}(2x,\frac{y}{2})& if \;0\le x<\frac{1}{2}\\ (2x-1,\frac{y}{2}+\frac{1}{2})& if \frac{1}{2}\le x<1\end{array}\right.$$
  - Iteration: The transformation is applied iteratively to achieve the encryption.

#### Hybrid combinations

- Henon + Arnold: The image is first encrypted using the Henon map, followed by the ACM.
- Henon + Baker: The image is first encrypted using the Henon map, followed by the Baker map.
- Arnold + Baker: The image is first encrypted using the ACM, followed by the Baker map.

#### Encryption and embedding process

- Image Encryption: The secret images were encrypted using three different chaotic maps: Henon, Arnold, and Baker. Additionally, hybrid combinations of these maps were tested.
- Image-to-Audio Conversion: The encrypted images were converted into audio-like signals using the ISTFT.
- Embedding Process: The converted image signals were embedded within the high frequencies of the cover audio signals using DWT and IDWT.

#### Performance evaluation metrics

- Randomness Test: The randomness of the resulting encrypted image was tested using the National Institute of Standards and Technology (NIST) statistical test suite.
- Image Quality Metrics: The quality of the recovered image was evaluated using Peak Signal-to-Noise Ratio (*PSNR*), Structural Similarity Index Measure (*SSIM*), correlation, Number of Pixels Change Rate (*NPCR*), Maximum Deviation Measuring Factor (*MDMF*), and Irregular Deviation Measuring Factor (*IDMF*).
- Audio Quality Metrics: The quality of the stego-audio signal was assessed using *PSNR*, Signal-to-Noise Ratio (*SNR*), Segmental SNR (*SNR*_*seg*_), Mean Squared Error (*MSE*), Spectral Distortion (*SD*), and Log-Likelihood Ratio (*LLR*).

Tables 1 and 2 show the quality metrics of the image and audio signals recovered from the proposed technique, which embeds the encrypted image within a cover audio signal. We used different chaotic maps for encrypting the target image before embedding it within the cover audio signal. We applied various types of chaotic image encryption techniques such as Henon, Arnold, and Baker chaotic maps, and different hybrid image encryption techniques such as Henon + Arnold, Henon + Baker, and Arnold + Baker chaotic maps. The image quality is assessed using *PSNR*, *SSIM*, correlation, *NPCR*, *MDMF*, and *IDMF*. The randomness of the resulting encrypted image was also tested using the NIST test. The audio signal quality is assessed using the *PSNR*, *SNR*, *SNR*_*seg*_, *MSE*, *SD*, and *LLR*.

By observing the results in Table 1, we note that the quality of the recovered image is affected by adding the image encryption step, but it is added for the sake of increasing the system security and its resistance against any external attack. We also note that by comparing the performance in several encryption cases, when the Arnold or Baker maps are used alone or in combination to encrypt the image, the recovered image quality is acceptable. As for the Henon map, it gives lower quality compared to the rest of the cases. It was also noted that the Henon chaotic map gives the highest PSNR, SSIM, and NPCR for the encrypted image, which indicates that the technique can resist different attacks, effectively.

Table 2 shows that in every instance of image encryption, the audio signal quality remains unaffected by the secret image being hidden inside it. This indicates that the proposed concealment technique has high robustness and strength in preserving the carrier signal.

Table 3 presents the outcomes of concealing the encrypted image through the individual utilization of Henon, Arnold, and Baker chaotic maps, as well as the hybrid combination of two chaotic maps. The results indicate that employing a hybrid structure of chaotic maps demands a comparatively extended duration; however, it notably enhances the security of the system and amplifies the challenge facing attackers.

### Comparison with recent literature

Table 4 presents a comparative study of prevalent audio steganography methodologies. As evident from the table, the LSB technique demonstrates an optimal performance in terms of image recovery quality; however, it yields unsatisfactory results for recovered audio signal quality. Conversely, the phase coding technique exhibits favorable outcomes for images, albeit registering the poorest results for audio among other methods. Moreover, the spread spectrum technique delivers commendable image quality and excels in audio quality performance. The STFT technique showcases proficient performance in both image and audio domains. When ranking these techniques based on image quality preservation, the LSB emerges as the most effective, followed by phase coding, spread spectrum, and STFT. Conversely, concerning audio recovery quality, the spread spectrum ranks highest, trailed by STFT, LSB, and phase coding.

It is noteworthy that upon employing the Henon, Arnold, and Baker chaotic maps for image encryption alongside all aforementioned techniques, it was observed that LSB, phase coding, and spread spectrum could not retrieve the decrypted hidden image. Only, the proposed technique demonstrates proficiency in decrypting the concealed image.

NIST tests are primarily intended for evaluating the randomness of random number generators. They can be adapted to assess the randomness of encrypted data, including images. They offer several statistical tests for assessing the randomness or quality of cryptographic algorithms, including those used in encryption^33^. Here is a generalized approach that could be considered to evaluate the randomness of an encrypted image using NIST test:

1. Encrypt the Image: Use your chosen encryption algorithm to encrypt the image data.
2. Extract Bit Sequence: Convert the encrypted image into a sequence of bits, depending on the encryption algorithm and mode used.
3. Apply NIST Statistical Tests: Use the NIST Statistical Test Suite to analyze the extracted bit sequence for randomness. This test includes many tests, such as the frequency test, block frequency test, runs test, longest run of one’s test, binary matrix rank test, serial test, and cumulative sums test.
4. Interpret Results: Evaluate the results of the statistical tests to determine if the encrypted image passes or fails the randomness tests as shown in Table 5. A passing result indicates that the encrypted image exhibits characteristics of randomness, while a failing result suggests the presence of patterns or biases that could compromise the security of the encryption.

**Table 5:** NIST test results for the randomness of the encrypted image.

Test name	Result value (*P*-value)	Status
Frequency (Monobit) test	0.26080	Passed
Frequency test within a block	0.28084	Passed
Runs test	0.63396	Passed
Test for the longest run of one’s in a block	0.92929	Passed
Binary matrix rank test	0.32378	Passed
Serial test	*P*-value 1: 0.10272 *P*-value 2: 0.03333	Passed
Cumulative sums (Custom) test	*P*-value Forward: 0.01278 *P*-value Reverse: 0.15002	Passed

### Evaluation

The efficiency of the suggested technique can be evaluated by:

1. Estimating the recovered image quality.
2. Estimating the encrypted image quality.
3. Estimating the stego-audio signal quality.

The simplest means to define image *PSNR* is with the *MSE*.

8 $$MSE_{I} = \frac{1}{{N_{r} \times N_{c} }}\mathop \sum \limits_{i = 1}^{{N_{r} }} \mathop \sum \limits_{j = 1}^{{N_{c} }} \left( {p_{ij} - q_{ij} } \right)^{2}$$

9 $$PSNR_{I} = 10.{\text{log}}_{10} \left( {\frac{{MAX_{I}^{2} }}{{MSE_{I} }}} \right) = 20.{\text{log}}_{10} \left( {\frac{{MAX_{I} }}{{\sqrt {MSE_{I} } }}} \right)$$

where *N*_*r*_ and *N*_*c*_ are the numbers of rows and columns in the secret image, and in the *i*th row and *j*th column, there exist the target image pixel *p*, and the recovered image pixel *q*. The Maximum Pixel Intensity value (*MAX*_*I*_) of the image is 255.

10 $$SSIM_{I} (s,t) = \frac{{(2\mu_{s} \mu_{t} + K_{1} )(2\sigma_{st} + K_{2} )}}{{(\mu_{s}^{2} + \mu_{t}^{2} + K_{1} )(\sigma_{s}^{2} + \sigma_{t}^{2} + K_{2} )}}$$

The images *x* and *y* means, standard deviations, and cross-covariance are provided as $\mu_{s} ,\mu_{t} ,\sigma_{s} ,\sigma_{t}, \text{and}\,\sigma_{st}$, respectively.

The relationship between two variables is measured by the correlation coefficient. If the original and the recovered images are significantly dependent on one another, they have a high correlation. In this instance, the recovered image matches the original one, demonstrating the effectiveness of the steganography technique in restoring the original image details. Therefore, high correlation coefficient values indicate the efficacy of the recommended methodology. The correlation coefficient can be calculated as follows^34^:

11 $${\text{Correlation Coefficient}} = \frac{{{\text{cov}} (x,y)}}{{\sigma_{x} \sigma_{y} }} = \frac{{\sum\nolimits_{i = 1}^{N} {(x_{i} - E(x))} (y_{i} - E(y))}}{{\sqrt {\sum\nolimits_{i = 1}^{N} ({x_{i} - E(x))^{2} } } \sqrt {\sum\nolimits_{i = 1}^{N} {y_{i} - E(y))^{2} } } }}$$

where ($E(x) = \frac{1}{N}\sum\nolimits_{i = 1}^{N} {x_{i} }$) and the grayscale pixel values of the original and recovered images are *x* and *y*, respectively.

The most popular measure for evaluating how resilient image encryption algorithms or ciphers are against differential attacks is the NPCR, $N(C^{1} ,C^{2} )$^35^. We think about the ciphertext images that appear before and after a plaintext image is changed by only one pixel. The $C^{1} (i,j)$, and $C^{2} (i,j)$ indicate the values of pixels at grid (*i*, *j*) in the encrypted images $C^{1}$ and $C^{2}$.

12 $$D(i,j) = \left\{ {\begin{array}{*{20}c} 0 & {if} & {C^{1} (i,j) = C^{2} (i,j)} \\ 1 & {if} & {C^{1} (i,j) \ne C^{2} (i,j)} \\ \end{array} } \right.$$

13 $$N(C^{1} ,C^{2} ) = \sum\limits_{i,j} {\frac{D(i,j)}{T} \times 100\% }$$

In the given scenario, the symbol *D* represents a bipolar array, and the symbol *T* denotes the total number of pixels.

The MDMF measures the maximum deviation between the original and the recovered images^36^. The IDMF is based on how much the deviation caused by encryption on the original image is irregular^37^. It gives attention to each pixel value and the deviation caused at every location of the original image. The higher the IDMF value is, the better the encryption algorithm^38^.

The audio signal *SNR*^39^ can be calculated as follows:

14 $$SNR_{S} = 10{\text{log}}_{10} \frac{{\mathop \sum \nolimits_{i = 1}^{{N_{s} }} u^{2} \left( i \right)}}{{\mathop \sum \nolimits_{i = 1}^{{N_{s} }} \left( {u\left( i \right) - v\left( i \right)} \right)^{2} }}$$

where *N*_*s*_ is the total number of samples, *i* is the sample index, *u*(*i*) is the original audio signal, and *v*(*i*) is the recovered audio signal.

The average of *SNR* over short segments is known as *SNR*_*seg*_. For a waveform, where the objective is to produce the same audio sound rather than the waveform itself, *SNR*_*seg*_ performance is a reasonable predictor of audio signal quality. One way to calculate the *SNR*_*seg*_ is as follows:

15 $$SNR_{seg} = \frac{10}{G}\mathop \sum \limits_{g = 0}^{G - 1} {\text{log}}_{10} \mathop \sum \limits_{{i = L_{s} g}}^{{L_{s} g + L_{s} - 1}} \left( {\frac{{u^{2} \left( i \right)}}{{\left( {u\left( i \right) - v\left( i \right)} \right)}}} \right)^{2}$$

where *G* is the number of segments in the audio signal and *L*_*s*_ is the segment length.

In the frequency domain, the *SD* is estimated. It illustrates how the recovered signal spectrum deviates from the original signal spectrum. It is described as follows:

16 $$SD = \frac{1}{G}\sum\limits_{g = 0}^{G - 1} {\sum\limits_{{i = L_{s} g}}^{{L_{s} g + L_{s} - 1}} {\left| {V_{u} (i) - V_{v} (i)} \right|^{{}} } }$$

where the spectra of the original and recovered time-domain audio segments are $V_{u} (i)$ and $V_{v} (i)$, respectively. They are measured in dB. The smaller the SD is, the better the proposed technique performance.

The basic idea of the *LLR* is the notion that an all-pole p-th order Linear Predictive Coding (LPC) model can be used to represent an audio segment. The more effectively the steganography technique operates, the closer the *LLR* to zero. The *LLR* measure is defined as^40^:

17 $$LLR = \left| {\log \left( {\frac{{\vec{a}_{u} \overline{R}_{v} \vec{a}_{u}^{T} }}{{\vec{a}_{v} \overline{R}_{v} \vec{a}_{v}^{T} }}} \right)} \right|$$

where $\vec{a}_{u}$ is the LPC coefficient vector for the original audio, $\vec{a}_{v}$ is the LPC coefficient vector for the recovered audio, and $\overline{R}_{v}$ is the distorted audio signal autocorrelation matrix. The performance of the *LLR* is restricted by the distortion situations where this hypothesis is valid,^41^ because it is predicated on the hypothesis that audio signals are well captured using an all-pole model. If the original audio is transferred through a voice communication system that dramatically alters the statistics of the original audio signal, this hypothesis may not be accurate.

## Conclusion and future work

This research work presented a novel audio steganography technique that combines chaos Henon, Baker, and Arnold encryption maps, with the STFT steganography to enhance the security and imperceptibility of hidden information, making the whole process resilient against potential attacks. The suggested technique has proven to be effective by the experimental findings, which establish its reputation as a reliable means of secret and secure audio signal communication that preserves the original information. The effectiveness of the proposed technique was evaluated through assessments of both the encrypted and recovered image quality, alongside evaluations of audio signal quality using different evaluation metrics. Furthermore, the technique was compared to other established approaches. Nonetheless, certain constraints were encountered, including the evaluation reliance on modest dataset sizes. Future work may include real-world deployment scenarios and performance evaluations under various practical constraints that can be explored to assess the technique applicability in different contexts. We can also study the realization of the proposed technique via FPGA. In addition, we will conduct a comparative study of different techniques used to include images in the audio signals. In addition, we will study the transmission of sound through a real communication system such as OFDM. We will also study the use of different enhancement techniques to improve the quality of the extracted signals. In addition, we will expand the scope of work on larger and different datasets and evaluate how this affects the performance of the methodology.

## Data Availability

The datasets generated and/or analyzed during the current study are not publicly available but are available from the corresponding author on reasonable request.

## References

[1] Arora, N. Types and tools of steganography. *Int. J. Res. Appl. Sci. Eng. Technol.* **10**(6), 2049–2053 (2022).

[2] Mathivanan, P. & Balaji Ganesh, A. ECG steganography using Base64 encoding and pixel swapping technique. *Multimed. Tool Appl.* **82**(10), 14945–14962 (2023).

[3] Nisha, O. T., Hossain, M. S., & Rahman, M.Audio steganography with intensified security and hiding capacity, (Doctoral dissertation, Hajee Mohammad Danesh Science and Technology University), 2023.‏

[4] Gera, A., & Vyas, V. Encrypted, compressed, and embedded text in audio WAV file using LSB audio stenography, Sustainable computing: transforming industry 4.0 to Society 5.0. Springer, Cham, pp. 291–305, 2023.‏

[5] Murhty, G. K. & Kanimozhi, T. Methodologies in steganography and cryptography–review. *Modn. Approaches Mach. Learn. Cognit. Sci.: A Walkthrough* **4**, 205–214 (2024).

[6] Saberi Kamarposhti, M., Ghorbani, A. & Yadollahi, M. A comprehensive survey on image encryption: Taxonomy, challenges, and future directions. *Chaos, Solitons & Fractals* **178**, 114361 (2024).

[7] Mathivanan, P. & Maran, P. A color image encryption scheme using a customized map. *Imag. Sci. J.* **71**(4), 343–361 (2023).

[8] Mathivanan, P. & Maran, P. Correction to Color image encryption based on novel Kolam scrambling and modified 2D logistic cascade map (2D LCM). *J. Supercomput.* **80**(3), 4420–4422 (2024).

[9] Parthasarathy, V. D. & Visvalingam, K. Healthcare data security in cloud storage using light weight symmetric key algorithm. *Int. Arab J. Inform. Technol.* **21**(1), 57–66 (2024).

[10] Zhang, B. & Liu, L. Chaos-Based image encryption: Review application, and challenges. *Mathematics* **11**(11), 1–39 (2023).

[11] Yaghoobi M. A simple and robust approach for image hiding using a chaotic logistic map. in IEEE International conference on advanced computer theory and engineering, pp. 623–627, 2008.‏

[12] Lin, C. C., Wang, P. Y., Lin, Y. H. Huang, H. C., & Saberikamposhti, M. Visible watermark removal with deep learning technology. in IEEE 6th international symposium on computer, consumer and control (IS3C), pp. 186–189, 2023.‏

[13] Al-Juaid, N. & Gutub, A. Combining RSA and audio steganography on personal computers for enhancing security. *SN Appl. Sci.* **1**, 1–11 (2019).

[14] Bharti, S. S., Gupta, M. & Agarwal, S. A novel approach for audio steganography by the processing of amplitudes and signs of secret audio separately. *Multim. Tool Appl.* **78**(16), 23179–23201 (2019).

[15] Abdulrazzaq, S. T., Siddeq, M. M. & Rodrigues, M. A. A novel steganography approach for audio files. *SN Comp. Sci.* **1**, 1–13 (2020).

[16] Hameed, A. S. A high secure speech transmission using audio steganography and duffing oscillator. *Wirel. Pers. Commun.* **120**(1), 499–513 (2021).

[17] Rakshit, P., Ganguly, S., Pal, S., Aly, A. A. & Le, D. N. Securing technique using pattern-based LSB audio steganography and intensity-based visual cryptography. *Comput., Mater. Continua* **67**(1), 1207–1224 (2021).

[18] Abood, E. W., Abduljabbar, Z. A., Al-Sibahee, M. A., Hussain, M. A. & Hussien, Z. A. Securing audio transmission based on encoding and steganography. *Indones. J. Electr. Eng. Comput. Sci.* **22**(3), 1777–1786 (2021).

[19] Paniker, P. V. Enhancing data security using text cryptography and multimedia steganography, (Doctoral dissertation, Dublin, National College of Ireland), 2022.‏

[20] Phipps, A., Ouazzane, K. & Vassilev, V. Securing voice communications using audio steganography. *Int. J. Comput. Netw. Inform. Secur. (IJCNIS)* **14**(3), 1–18 (2022).

[21] Alhassan, S., Daabo, M. I. & Armah, G. K. Twin K-shuffle based audio steganography. *Asian J. Eng. Appl. Technol.* **11**(1), 1–4 (2022).

[22] Marszałek, P. & Bilski, P. Steganography in audio files: COTS software analysis. *Int. J. Electr. Telecommun.* **69**(1), 121–126 (2023).

[23] Arian, M., Saberi Kamarposhti, M., Broumandnia, A. A new method for image encryption using DNA sequences and hyperchaos. in International conference on mechanical, manufacturing, and process plant engineering, Springer Nature, Singapore, pp. 371–378, 2023.

[24] Sahlabadi, M., Saberikamarposhti, M., Muniyandi, R. C., & Shukur, Z., Using cycling 3D chaotic map and DNA sequences for introducing a novel algorithm for color image encryption. in IEEE International conference on cyber resilience (ICCR), pp. 1–7, 2022.‏

[25] Al-Abass, S. A. A., Hayder, M. A., Abdul-Sattar, Z. S., Rasool, O. H. & Hasan, M. A. Color image encryption and decryption by using chaotic Baker map bit interleaver. *Int. Res. J. Eng. Technol. (IRJET)* **4**(5), 382–385 (2017).

[26] Ayoup, A. M. *et al.* Cancellable multi-biometric template generation based on Arnold cat map and aliasing. *Comput., Mater. Continua* **72**(2), 3687–3703 (2022).

[27] Abdallah, A. A. & Farhan, A. K. A new image encryption algorithm based on multi chaotic system. *Iraqi J Sci*10.24996/ijs.2022.63.1.31 (2022).

[28] Ibrahim, S. & Alharbi, A. Efficient image encryption scheme using Henon map, dynamic S-boxes and elliptic curve cryptography. *IEEE Access* **8**, 194289–194302 (2020).

[29] Saberi Kamarposhti, M., Sahlabadi, M., Lin, C. C. & Muniyand, R. C. Using 2D Henon map, cycling chaos, and DNA sequence for new secure color image encryption algorithm. *Arab. J. Sci. Eng.* **49**(3), 4125–4137 (2024).

[30] Ghorbani, A., Saberikamarposhti, M. & Yadollahi, M. Using ribonucleic acid (RNA) and Hénon map in new image encryption scheme. *Optik* **259**, 168961 (2022).

[31] Nasr, M. A. *et al.* Efficient information hiding in medical optical images based on piecewise linear chaotic maps. *J. Opt.* **52**(4), 1852–1866 (2023).

[32] El-Hoseny, H. M., Ahmed, H., Kazemain, H., Abd El-Samie, F., & Abbas, A. M., Digital image encryption in transform domains, MSc thesis, Department of electronic and electrical communication engineering, faculty of electronic engineering, Menoufia University 2014.‏

[33] Sabah, A., Hameed, S. & Maisa’a-Abid-Ali, K. Key Generation based on Henon map and Lorenz system. *Al-Mustansiriyah J. Sci.* **31**(1), 41–46 (2020).

[34] Faragallah, O. S. Digital image encryption based on the RC5 block cipher algorithm. *Sens. Imag.: Int. J.* **12**, 73–94 (2011).

[35] Ozkaynak, F. Role of NPCR and UACI tests in security problems of chaos-based image encryption algorithms and possible solution proposals. in IEEE international conference on computer science and engineering (UBMK), pp. 621–624, 2017.

[36] Faragallah, O. S. *et al.* Efficient optical double image cryptosystem using chaotic mapping-based Fresnel transform. *Opt. Quant. Electr.* **53**(6), 1–26 (2021).

[37] Z. Yun-Peng, L. Wei, C. Shui-Ping, Z. Zheng-Jun, N. Xuan, & D. Wei-Di. Digital image encryption algorithm based on chaos and improved DES. in IEEE international conference on systems, man, and cybernetics, pp. 474–479, 2009.‏

[38] Amin, M., Faragallah, O. S. & Abd El-Latif, A. A. A chaotic block cipher algorithm for image cryptosystems. *Commun. Nonlin. Sci. Numer. Simul.* **15**(11), 3484–3497 (2010).

[39] Nasr, M., El-Rabaie, S., Abd El-Samie, F., El-Fishawy, A. & Abd-Elnaby, M. Efficient implementation of adaptive Wiener filter for pitch detection from noisy speech signals. *Menoufia J. Electr. Eng. Res. (MJEER)* **27**(1), 109–126 (2018).

[40] Abd El-Fattah, M. A., Dessouky, M. I., Diab, S. M. & Abd El-Samie, F. E. Adaptive Wiener filtering approach for speech enhancement. *Ubiquit Comput. Commun. J.* **3**(2), 23–31 (2008).

[41] Hashim, M. M., Rahim, M. S. M., John, F. A., Taha, M. S. & Hamad, H. S. Performance evaluation measurement of image steganography techniques with analysis of LSB based on variation image formats. *Int. J. Eng. Technol.* **7**(4), 3505–3514 (2018).

